# Night shift work and breast cancer risk: A cohort study based on payroll and survey data from Finland

**DOI:** 10.5271/sjweh.4309

**Published:** 2026-07-01

**Authors:** Rahman Shiri, Päivi Vanttola, Jenni Ervasti, Aki Koskinen, Johnni Hansen, Mikko Härmä

**Affiliations:** 1 Finnish Institute of Occupational Health, Helsinki, Finland.; 2 Danish Cancer Institute, Danish Cancer Society, Copenhagen, Denmark.

**Keywords:** chronotype, fatigue, psychological distress, shift work schedule, sleep disturbance

## Abstract

**Objective:**

This study aimed to (i) investigate associations between different characteristics of exposure to shift work and breast cancer risk and (ii) identify effect modifiers.

**Methods:**

Comprising 42 379 women from the Finnish Public Sector cohort, this longitudinal study included data from survey responses, payroll-based working hour records, and national breast cancer registry data. Cause-specific Cox proportional hazards models were used to estimate hazard ratios (HR), adjusting for age, marital status, socioeconomic status, living with a child, lifestyle, and occupational factors. Stratified analyses and interaction tests were performed to assess effect modification.

**Results:**

Over an average follow-up of 9.8 years, 945 women were diagnosed with breast cancer. Among women aged ≥50 years, increased risk was associated with working ≥3 consecutive night shifts [hazard ratio (HR) 1.64, 95% confidence interval (CI) 1.22–2.21] and evening shifts (HR 1.28, 95% CI 1.03–1.59). Sleep problems and fatigue at work modified the association between night shift work and breast cancer risk, whereas psychological distress and diurnal preference did not. Permanent night shift workers reporting sleep problems (HR 1.76, 95% CI 1.01–3.09) or fatigue at work (HR 1.90, 95% CI 1.08–3.35) had a higher risk compared to day workers with similar symptoms.

**Conclusions:**

Women aged ≥50 years showed increased breast cancer risk for several shift work characteristics. Permanent night shift work was especially associated with increased risk among those reporting sleep problems or fatigue. These findings may help identify groups needing closer occupational health monitoring, though causal inference is limited by the observational design. Intervention studies are required to determine whether changes in work schedules or management of sleep problems and fatigue could reduce risk.

The International Agency for Research on Cancer (IARC) conducted comprehensive reviews of night shift work and human, experimental animals, and biological mechanism evidence in 2007 and 2019 (1, 2). It concluded that night shift work probably causes breast cancer. This overall evaluation was based on sufficient evidence in experimental animals for the carcinogenicity of alteration in the light–dark schedule. There was limited evidence in epidemiology because bias could not be reasonably ruled out. The most consistent epidemiologic evidence came from case–control rather than cohort studies (1, 2). The IARC working group considered a pooled case–control study based on >6000 breast cancer cases particularly informative (3). Unlike most other studies, this study observed an increased risk for breast cancer primarily in pre-menopausal compared to post-menopausal women (3). This finding was not fully supported by data from the most influential cohorts of rotating night shifts – the Nurses' Health studies (N=~200 000) (4) – or our previous Finnish Public Sector (FPS) cohort-based study (5). Furthermore, some other large cohort studies did not show associations between night shift work and breast cancer risk (6–8). It has been suggested that more comprehensive and detailed exposure assessments in case–control versus cohort studies may at least partly explain the difference in results (2).

Over ten epidemiologic studies on night work and breast cancer have been published after the IARC evaluation but have neither contributed substantially to strengthening the IARC evaluation nor offered further insight in the impact of menopausal status or chronotype (ie, diurnal preference) (9). Misclassification of night shift work may occur in both case–control and cohort studies, whereas differential recall bias may remain an Achilles heel especially for case–control studies. The use of objective register-based payroll data to access working hours may exclude this bias. A few studies have utilized such data, including notable investigations from Denmark (10) and Sweden (11) focusing on healthcare workers. However, these earlier payroll-based studies primarily examined the short-term effects of night shift work, characterized by limited follow-up periods and a lack of information regarding cumulative lifetime exposure to shift work before baseline. The discrepancy observed in earlier studies, along with the need for more prolonged exposure and follow-up periods, highlights the importance of further research into the association between night shift work and breast cancer.

Previous longitudinal studies as well as case–control studies have shown that potential confounders, such as smoking, body mass index, physical activity, and alcohol consumption, do not seem to substantially alter the association between night shift work and breast cancer risk (7, 8). However, the potential role of other factors – such as sleep duration, sleep quality, psychological distress, and fatigue – on breast cancer risk remains insufficiently understood (12, 13). Insomnia and fatigue are common among night shift workers (14) and can be signs of individual vulnerability or circadian disruption (15). A previous study from Finland showed that night shift workers with longer sleep duration (>8 hours) had a higher risk of breast cancer than day workers (16). However, another study found that the risk does not differ by hours of sleep (<7 versus >7 hours) or taking medications to sleep (8). Poor sleep quality, psychological distress, and fatigue are prevalent among breast cancer patients (12, 13). However, their potential role in breast cancer risk warrants further investigation.

This study aimed to examine the association between shift work – with and without night shifts, defined using multiple exposure characteristics – and the incidence of breast cancer among female public sector workers, including potential differences by age, chronotype (ie, diurnal preference), sleep problems and fatigue.

## Methods

### Data sources and linkage

We linked payroll-based daily working hour records from the Working Hours in Finnish Public Sector (WHFPS) cohort (17) with survey data and national breast cancer registry data from the Finnish Public Sector (FPS) cohort (18). Data linkage was performed using the unique personal identification number assigned to all Finnish residents.

### Population

The dynamic study cohort consisted of 42 379 women who had: (i) entered the FPS/WHFPS cohort in 2008–2018, (ii) responded to ≥1 FPS questionnaire at baseline (year of entry), (iii) worked ≥30 days during the baseline year, and (iv) ≥1 year of payroll data available prior to the start of the cancer follow-up.

The FPS cohort included employees of healthcare and social services from 11 municipalities and five hospital districts. The study population comprised two sub-cohorts: the hospital cohort (survey years: 2008, 2012, 2014, 2015, and 2017), and the 10-town cohort (survey years: 2008, 2012, 2014, 2016, and 2018). The average response rate across FPS surveys was 70%.

Women diagnosed with breast cancer prior to baseline and during the exposure period were excluded (N=592). Data on chronotype were available for 22 481 participants and information on fatigue at work for 22 884 participants.

### Outcome

Data on breast cancer cases were obtained from the Finnish Cancer Registry using the International Classification of Diseases for Oncology (ICD-O) codes C50.0– C50.9. The data covered diagnoses made in 1980–2022.

### Exposure variables

A night shift was defined as working ≥3 hours during 23:00–06:00 hours, while an evening shift involved work during 06:00–23:00 hours and was not defined as a night shift. A morning shift started at 06:00–08:00 hours and ended before 18:00 hours and was not defined as a night shift (19). Non-day shifts included evening and night shifts.

Using 2008–2018 payroll data at study entry, we derived several baseline indicators of shift and night work exposure. These included the annual number of: any shifts; non-day shifts; morning shifts; evening shifts, night shifts lasting ≥8, 10, or 12 hours; and quick returns following the last shift (<11 hours) and the last night shift (<28 or 48 hours). The annual frequency of sequences with any, ≥3, or ≥5 consecutive night shifts and the yearly ratio of night to total shifts were also included (20). Working-hour characteristics were not suitable to be analyzed as continuous variables because their distributions were highly zero-inflated, with a large proportion of participants having no exposure. To allow examination of potential dose–response relationships, we divided the exposed participants into two groups. Except for the number of any shift work and morning shifts, all exposure variables were therefore categorized into three levels: (i) no exposure (non-shift or non-night workers), (ii) exposure below the median among those exposed, and (iii) exposure above the median among those exposed. The median value was used to split the exposed group into two approximately equal-sized categories.

### Effect modifiers

The FPS survey collected information on age, sleep problems, fatigue, psychological distress and chronotype. Sleep problems were assessed using the 4-item Jenkins Sleep Scale, which evaluates: (i) difficulty falling asleep, (ii) waking during the night, (iii) difficulty staying asleep, and (iv) non-restorative sleep in the last 4 weeks (18). Participants who reported multiple insomnia symptoms at baseline were classified according to their most frequent symptoms. Sleep problems were dichotomized as: no (0–1 night/week) and yes (2–7 nights/week) (21). Information on fatigue in the past four weeks was collected only among participants of the hospitals cohort (14), using two items: (i) "How often have you experienced fatigue during work hours?" and 2) "How often have you experienced fatigue during free days?" Response options were: (i) not at all, (ii) 1–3 times per month, (iii) about once per week, (iv) 2–4 times per week, (v) 5–6 times per week, and (vi) nearly every day. Fatigue during work or free days was dichotomized as experiencing fatigue at least once per week versus never or only 1–3 times per month. Based on our earlier results, both fatigue at work and fatigue during free days were associated with night shift work (14). Psychological distress was assessed using the 12-item General Health Questionnaire (GHQ-12) (22). Scores were 0–12, with ≥3 classified as indicative of psychological distress. Information on chronotype was taken from the year 2015 (hospital cohort) or 2018 (10-town cohort) for those entering the cohort on that year or replying to the survey on those years but entering the cohort earlier due to an earlier survey. Chronotype was assessed using one item from the Diurnal Type Scale (23): "Do you think you are a morning person or an evening person?" The response options were definite morning type, somewhat morning type, somewhat evening type, and definite evening type. Due to limited statistical power, we combined these categories into two groups types: morning (definite morning + somewhat morning) and evening (somewhat evening + definite evening).

### Covariates

The FPS data includes information on age, marital status, socioeconomic status (upper white collar, lower white collar, skilled blue collar, and other blue collar), living with a child, height, weight, smoking habits, alcohol consumption, physical activity, job demands, job control, and worktime control (seven items) (24). Body mass index (BMI) was calculated based on self-reported height and weight. Smoking status was coded as reported (never or ever [current or former] smoker. Physical activity during commuting or leisure time over the past 12 months was measured using the four intensity levels: (i) walking, (ii) brisk walking, (iii) jogging, and (iv) running. We computed a metabolic equivalent (MET) index by multiplying the MET value of each activity intensity by the time spent on that activity and adding up the MET of the four activities. We assessed alcohol consumption by determining the weekly intake of beer and wine, along with the monthly consumption of spirits. We then converted them to equivalent units of alcohol, with each unit representing 12 grams of alcohol consumed per week. Job demands were measured using 5 items and job control was measured using 9 items (24).

### Statistical analysis

This prospective study of incident breast cancer of maximum 14-year follow-up started in January 2009 and ended in December 2022. We estimated the risk of breast cancer associated with morning, evening and night work. We also examined a dose–response association between breast cancer and the number of any shift; non-day shifts; morning shifts; evening shifts; night shifts of ≥8, 10, or 12 hours; any, ≥3, or ≥5 consecutive night shifts; and the ratio of night shifts to all shifts.

To identify effect modifiers, subgroup analyses were performed based on participants’ age at study entry (<50 versus ≥50 years) as a proxy for menopausal status, on the presence or absence of insomnia, fatigue, and psychological distress, and chronotype. Multiplicative effect modification was assessed by including interaction terms between the type of shift work and sleep problems or fatigue at work in analyses of the total study population.

Given the dynamic cohort design of the FPS study, the existence of censored data, and the presence of a competing event, the Cox proportional hazards model was used to analyze the data (25). Within the framework of the Cox model, a cause-specific hazard model was employed (26). We calculated person-time for each participant from end of exposure period up to the date of death, cancer diagnosis or the end of follow-up on 31 December 2022. We accounted for the clustering effect of work units as the intraclass correlation coefficient for units exceeded the threshold of 0.05 (27) at 0.09. At higher organizational levels (municipality and hospital district), no additional clustering effect was observed. We tested the proportional hazards assumption using scaled Schoenfeld residuals against time and the log-log plots (25). These tests confirmed that the effect of exposures on breast cancer risk remained constant over time. Hazard ratios (HR) were adjusted for age, marital status, socioeconomic status, living with a child, tobacco smoking, alcohol consumption, BMI, physical activity during leisure time, job demands, job control, and worktime control. The analysis was conducted using Stata software, version 18 (Stata Corp, College Station, TX, USA).

## Results

### Descriptive characteristics

Among the 42 379 women included in the study, 945 developed breast cancer during a follow-up period of up to 14 years, with a mean of 9.8 (standard deviation 3.1) years. The follow-up duration was <6 years for 3.6% of participants and <8 years for 20.1%. The average follow-up time was 9.7 years among women aged <50 years and 10.0 years among those aged ≥50 years. At baseline, 65% of participants were <50 years, while 35% were ≥50 years (table 1). Based on survey responses, 27% of participants reported working in shifts that included night work or permanent night shifts. Additionally, 21% were classified as obese (BMI >30 kg/m^2^), and 37% were either current or former smokers. Median leisure-time physical activity was 27.5 MET per week, with an interquartile range from 15.8 (25^th^ percentile) to 50.0 (75^th^ percentile). Fatigue at work at least once per week was reported by 60% of participants, and 57% reported psychological distress.

**Table 1:** Baseline characteristics of the study population (N=42 379).

Characteristic		%
Age (years)		
	17–29	16
	30–39	22
	40–49	27
	50–59	28
	60–72	7
Socioeconomic status		
	Upper white collar	60
	Lower white collar	21
	Skilled blue collar	15
	Other blue collar	5
Ever smoking		37
Body mass index		
	Overweight (body mass index 25.0–29.9 kg/m^2^)	30
	Obesity (body mass index >30.0 kg/m^2^)	21
Chronotype		
	Morning	52
	Evening	48
Type of shift work		
	Regular day work	44
	Shift work without night shifts	27
	Shift work with night shifts	25
	Permanent night work	2
	Other irregular work	3
Sleep problems (Jenkins)		51
Psychological distress		57
Fatigue at work at least once per week		60
Fatigue during free time at least once per week		45

Among the total sample, an average of 13.5% of all annual shifts were evening, and 6.8% were night shifts. Additionally, about 10% of all shifts were scheduled with less than 11 hours of rest between shifts.

### Associations between working-hour characteristics and breast cancer

In the total sample, no working-hour characteristics showed an association with breast cancer (table 2).

**Table 2:** The associations of shift and night work characteristics at baseline with breast cancer risk in the total sample. [HR=hazard ratio; CI=confidence interval.]

Characteristic at baseline		Sample	Cancer cases	Age–adjusted			Multivariable *	
				HR	95% CI		HR	95% CI
Type of shift work, self–reported								
	Regular day work	18 116	449	1			1	
	Shift work without night shifts	11 194	242	0.93	0.80–1.09		0.99	0.83–1.17
	Shift work with night shifts	10 161	197	0.92	0.78–1.09		0.92	0.76–1.12
	Permanent night work	782	22	1.23	0.82–1.86		1.07	0.67–1.73
	Other irregular work	1096	21	0.69	0.45–1.06		0.66	0.40–1.08
Number of non–day shifts								
	None	14 015	343	1			1	
	Below median	14 035	301	0.92	0.79–1.08		0.94	0.79–1.11
	Above median	14 048	296	1.00	0.86–1.17		0.98	0.83–1.17
Number of morning shifts								
	Low	14 044	279	1			1	
	Medium	14 014	296	0.97	0.82–1.14		0.99	0.83–1.18
	High	14 040	365	1.10	0.94–1.28		1.10	0.93–1.31
Number of evening shifts								
	None	19 480	479	1			1	
	Below median	11 315	227	0.92	0.78–1.08		0.96	0.80–1.14
	Above median	11 303	234	1.02	0.88–1.20		1.01	0.85–1.21
Number of night shifts >8 hours								
	None	28 626	678	1			1	
	Below median	6732	137	1.01	0.83–1.21		0.95	0.77–1.17
	Above median	6740	125	0.99	0.82–1.20		0.97	0.78–1.21
Number of night shifts >10 hours								
	None	30 481	716	1			1	
	Below median	5811	121	1.04	0.87–1.26		0.98	0.80–1.21
	Above median	5806	103	0.98	0.80–1.20		0.96	0.76–1.20
Number of night shifts >12 hours								
	None	36 217	839	1			1	
	Below median	2924	50	0.90	0.68–1.19		0.86	0.63–1.16
	Above median	2957	51	0.90	0.69–1.18		0.91	0.69–1.21
Number of consecutive night shifts								
	None	28 592	678	1			1	
	Below median	6014	122	0.92	0.76–1.12		0.85	0.69–1.06
	Above median	7492	140	1.07	0.89–1.28		1.06	0.87–1.30
>3 consecutive night shift								
	No	34 297	809	1			1	
	Yes	7801	131	0.97	0.81–1.17		0.97	0.78–1.19
>3 consecutive night shift								
	None	34 297	809	1			1	
	Below median	3768	55	0.83	0.63–1.09		0.76	0.56–1.04
	Above median	4033	76	1.11	0.88–1.41		1.17	0.90–1.52
>5 consecutive night shift								
	No	40 152	902	1			1	
	Yes	1946	38	1.12	0.81–1.55		1.11	0.76–1.62
Shift intervals of <11 hours								
	None	19 288	475	1			1	
	Below median	11 403	230	0.97	0.83–1.13		0.99	0.83–1.18
	Above median	11 407	235	0.91	0.78–1.06		0.93	0.78–1.10
Number of recovery periods <28h after the last night shift								
	None	37 540	855	1			1	
	Below median	2390	41	0.87	0.64–1.17		0.87	0.63–1.20
	Above median	2396	49	1.01	0.76–1.35		0.97	0.71–1.32
Number of recovery periods <48h after the last night shift								
	None	34 477	799	1			1	
	Below median	3841	68	0.90	0.71–1.14		0.85	0.66–1.11
	Above median	4008	78	1.00	0.79–1.25		0.93	0.72–1.19
Ratio of night shifts to all shifts								
	None	28 592	678	1			1	
	Below median	6750	137	1.00	0.83–1.21		0.94	0.77–1.16
	Above median	6756	125	0.99	0.82–1.20		0.97	0.78–1.21
Worktime control								
	Low	7841	161	1			1	
	Medium	10 851	189	0.92	0.75–1.13		0.87	0.70–1.07
	High	9870	199	1.14	0.93–1.40		1.13	0.92–1.40

* Adjusted for age, marital status, living with a child, socioeconomic status, smoking, alcohol consumption, body mass index, leisure–time physical activity, job demands, job control, and worktime control.

In the age-stratified analysis, among women aged <50 years (supplementary material, www.sjweh.fi/article/4309, table S1), those engaged in shift work involving night shifts (HR 0.73, 95% CI 0.54–0.996) or irregular shift work (HR 0.31, 95% CI 0.10–0.94) had a lower risk of breast cancer compared to day workers. Similarly, working non-day (HR 0.70, 95% CI 0.52–0.94) or evening (HR 0.71, 95% CI 0.52–0.96) shifts was associated with a reduced risk. In contrast, morning shifts were associated with an increased risk in this age group (HR 1.36, 95% CI 1.01–1.82).

Among women aged ≥50 years, working ≥3 consecutive night shifts (HR 1.64, 95% CI 1.22–2.21) was associated with a significantly increased risk of breast cancer. Although the risk also appeared elevated for working any (HR 1.28, 95% CI 0.99–165) or ≥5 (HR 1.46, 95% CI 0.95–2.26) consecutive night shifts, these estimates did not reach statistical significance. In addition, both non-day (HR 1.25, 95% CI 1.01–1.55) and evening (HR 1.28, 95% CI 1.03–1.59) shift work were associated with an increased risk of breast cancer in this older age group.

### Effect modification

Effect modification by sleep problems, fatigue at work, and chronotype are presented in tables 3–4 and supplementary tables S2–S4. Sleep problems and fatigue at work modified the association between night shift work and breast cancer risk. Specifically, in the stratified analysis, permanent night shift workers who reported sleep problems (HR 1.76, 95% CI 1.01–3.09) or experienced fatigue at work ≥1 per week (HR 1.90, 95% CI 1.08–3.35) showed a higher breast cancer incidence compared to day workers with sleep problems or fatigue. Similar patterns of elevated HR were observed among women aged both <50 and ≥50 years. Testing for multiplicative interaction showed a HR of 3.64 (95% CI 1.13–11.74) for the interaction between permanent night work and sleep problems (Wald test P=0.030) and 3.60 (95% CI 0.99–13.05) for the interaction between permanent night work and fatigue at work (P=0.051). In contrast, psychological distress (data not shown) and chronotype (supplementary Table S4) did not consistently modify the association.

**Table 3:** The association of type of shift work at baseline with breast cancer risk in women with sleep problems. [HR=hazard ratio; CI=confidence interval.]

Characteristic		Women without sleep problems					Women with sleep problems			
		Sample	Cancer cases	HR *	95% CI		Sample	Cancer cases	HR *	95% CI
**Total sample**										
Type of shift work, self–reported										
	Regular day work	8981	222	1			9118	227	1	
	Shift work without night shifts	5112	102	0.93	0.72–1.21		6051	139	1.04	0.82–1.31
	Shift work with night shifts	5029	100	0.96	0.73–1.26		5124	97	0.89	0.69–1.16
	Permanent night work	443	7	0.51	0.19–1.38		337	15	1.76	1.01–3.09
	Other irregular work	507	8	0.57	0.26–1.26		584	13	0.73	0.38–1.40
**Women <50 years**										
Type of shift work, self-reported										
	Regular day work	5636	91	1			5386	91	1	
	Shift work without night shifts	3295	35	0.78	0.51–1.20		3727	48	1.06	0.73–1.55
	Shift work with night shifts	3752	44	0.81	0.54–1.21		3621	33	0.67	0.43–1.04
	Permanent night work	293	2	0.28	0.04–1.20		224	6	1.81	0.78–4.23
	Other irregular work	311	1	0.22	0.03–1.56		329	2	0.39	0.10–1.50
**Women ≥50 years**										
Type of shift work, self–reported										
	Regular day work	3345	131	1			3732	136	1	
	Shift work without night shifts	1817	67	1.07	0.78–1.47		2324	91	1.05	0.78–1.41
	Shift work with night shifts	1277	56	1.12	0.76–1.63		1503	64	1.10	0.79–1.53
	Permanent night work	150	5	0.76	0.24–2.41		113	9	1.87	0.88–3.95
	Other irregular work	196	7	0.83	0.34–2.02		255	11	0.97	0.48–1.95

* Adjusted for age, marital status, living with a child, socioeconomic status, smoking, alcohol consumption, body mass index, leisure–time physical activity, job demands, job control, and worktime control.

**Table 4:** The association of type of shift work at baseline with breast cancer risk in women with fatigue at work. [HR=hazard ratio; CI=confidence interval.]

Characteristic		Women without fatigue at work					Women with fatigue at work			
		Sample	Cancer cases	HR *	95% CI		Sample	Cancer cases	HR *	95% CI
**Total sample**										
Type of shift work, self–reported										
	Regular day work	3869	132	1			5611	172	1	
	Shift work without night shifts	2133	56	0.89	0.64–1.24		3267	100	1.11	0.85–1.45
	Shift work with night shifts	2230	54	0.79	0.56–1.11		3342	89	1.01	0.77–1.34
	Permanent night work	244	3	0.52	0.17–1.60		309	13	1.90	1.08–3.35
	Other irregular work	269	6	0.75	0.34–1.63		371	7	0.63	0.30–1.32
**Women <50 years**										
Type of shift work, self–reported										
	Regular day work	2180	57	1			3067	56	1	
	Shift work without night shifts	1312	26	1.02	0.63–1.65		1922	31	1.18	0.73–1.91
	Shift work with night shifts	1576	25	0.72	0.44–1.18		2383	39	1.01	0.63–1.61
	Permanent night work	171	2	0.68	0.17–2.66		213	4	1.80	0.70–4.65
	Other irregular work	157	3	0.81	0.26–2.54		214	0	–	
**Women ≥50 years**										
Type of shift work, self–reported										
	Regular day work	1689	75	1			2544	116	1	
	Shift work without night shifts	821	30	0.82	0.52–1.29		1345	69	1.12	0.81–1.55
	Shift work with night shifts	654	29	0.93	0.57–1.50		959	50	1.05	0.74–1.49
	Permanent night work	73	1	0.39	0.05–2.75		96	9	2.12	1.05–4.29
	Other irregular work	112	3	0.65	0.21–2.07		157	7	0.96	0.46–2.02

* Adjusted for age, marital status, living with a child, socioeconomic status, smoking, alcohol consumption, body mass index, leisure–time physical activity, job demands, job control, and worktime control.

## Discussion

Overall, we observed little association between different characteristics of shift work compared to regular day work on breast cancer risk in the Finnish public sector. We observed an increased risk of breast cancer in the subgroup of permanent night shift workers who experienced sleep problems or fatigue at work ≥1 per week. This elevated risk was evident among women aged both <50 and ≥50 years at baseline. Additionally, among women aged <50 years, working morning shifts was associated with a higher risk of breast cancer. Among women aged ≥50 years, evening shifts and sequences of ≥3 consecutive night shifts were linked to an elevated risk. In contrast, psychological distress and chronotype did not appear to modify the relationship between night shift work and breast cancer risk.

Evidence on the association between consecutive night shifts and breast cancer risk among women aged ≥50 years remains limited and somewhat inconsistent (28, 29). While several studies have linked cumulative or frequent night shift work to elevated breast cancer risk (3), the specific impact of consecutive night shifts lack studies. Experimental studies have shown that working ≥3 consecutive night shifts per week is associated with hypermethylation of core circadian genes such as *ARNTL*, which may contribute to carcinogenesis (30). Additionally, a nested case–control study among Norwegian nurses found that working six consecutive night shifts for >5 years was associated with telomere shortening – a biomarker of cellular aging – and increased breast cancer risk (31).

These previous findings provide biological context for our results but should be regarded as hypothesis-generating rather than as evidence supporting a mechanistic interpretation of the observed interactions. As our analyses focused on effect modification rather than mediation, we cannot determine whether consecutive night shifts influence breast cancer through specific biological pathways. Further targeted epidemiological studies using designs suited for causal and mediation analysis are needed before conclusions regarding mechanisms can be drawn. Until such evidence is available, limiting the number of consecutive night shifts may be considered as a precautionary approach, rather than a causal inference, to reduce potential cancer risk (32).

Rather than providing evidence of an underlying causal pathway or mechanism, our findings suggest that night shift workers experiencing sleep problems or fatigue may represent subgroups at higher risk of breast cancer. To our knowledge, only one previous study has reported an increased breast cancer risk among night shift workers with longer sleep duration (>8 hours) (16). In contrast, another study found no significant differences in risk based on sleep duration (<7 versus >7 hours) or use of sleep medications (8). However, no prior studies have examined whether insomnia or fatigue at work modifies the association between night shift work and breast cancer risk. This is a notable gap, as night shift work is associated with sleep problems and fatigue during work and free days (14). Although these exposures are commonly discussed in relation to circadian disruption and hormonal dysregulation, in the present study they were analyzed as effect modifiers rather than mediators. Therefore, the interaction findings should be interpreted as identifying potentially susceptible subgroups, not as evidence supporting a specific biological mechanism. In our study, we found no evidence of interaction between night shift work and psychological distress in relation to breast cancer risk. Future research should further explore potential interactions using larger cohorts and more precise measures of stress and fatigue to better understand the multifactorial etiology of breast cancer.

Our finding of a lower breast cancer risk among shift workers aged <50 years at baseline contrasts with previous evidence from both case–control (3) and longitudinal (6) studies. A pooled analysis of five population-based case–control studies with complete work histories conducted in Germany, Spain, Canada, and Australia reported a significantly elevated risk of breast cancer among premenopausal women with previous night shift work (3). Similarly, earlier longitudinal studies have shown increased breast cancer risk among premenopausal women working night shifts (6, 33). In contrast, our study observed a reduced risk across all shift workers aged <50 years at baseline. However, these associations did not remain among women experiencing sleep problems or fatigue at work, who showed an increased risk. This pattern should be interpreted cautiously as it may reflect individual susceptibility, selection into or out of shift work, or healthy worker survivor effects. Some previous studies have also reported inverse associations. For example, a payroll-based study with incomplete work history from Denmark found a tendency of reduction in breast cancer risk among women who had worked night shifts in the past year (10). Similarly, another study with self-report data showed an inverse association between current night shift work and breast cancer risk (8). These findings highlight the need for more nuanced research that considers selection to shift work, duration and intensity of exposure, and individual vulnerability (1). Accordingly, references to circadian disruption and related biological pathways should be regarded as contextual and hypothesis-generating rather than as interpretations directly supported by the present study. Further investigation is warranted to clarify these associations and identify subgroups least and most at risk.

Our findings suggest that chronotype does not moderate breast cancer risk among shift and night workers. This result is consistent with findings from other cohort studies (8, 16), which reported no significant differences in breast cancer risk between individuals with morning and evening preferences. However, previous case–control studies found a significant association between night shift work and breast cancer exclusively among women with a morning chronotype (34, 35), suggesting that individuals with an evening chronotype may tolerate night work more effectively (36). These discrepancies may be partly attributed to differences in work schedules, age, and methodological differences. Retrospective designs, such as case–control studies, are more susceptible to recall bias, particularly in self-reported exposure data, which can inflate observed associations. Additionally, selection bias may arise if participation rates differ between cases and controls based on exposure status. These potential biases highlight the importance of using prospective designs in future research to clarify the role of chronotype in modifying the health effects of night shift work.

### Strengths and limitations

The present study benefits from a relatively large sample size and a substantial number of incident breast cancer cases, providing adequate statistical power for analyses across the total sample. The relatively high baseline response rate and long follow-up period further strengthen the reliability of the findings. Exposure assessment was enhanced using both self-reported data on work demands, sleep and lifestyle habits at baseline, and objective payroll records. The payroll data enabled detailed quantifications of night shift exposure, including characteristics such as the frequency of consecutive night shifts, and recovery periods following night shifts.

However, several limitations should be acknowledged. First, the follow-up period was shorter for participants who entered the study in later years, which may limit the ability to detect long-term effects in this subgroup. However, only 0.95% of participants had <4 years follow-up. Among women aged <50 years, the proportion was 0.47%, compared with 1.83% among those aged ≥50 years. Second, exposure to ≥5 consecutive night shifts was relatively rare, resulting in limited statistical power to detect associations in this category. Third, the study lacked sufficient statistical power to examine whether sleep problems or fatigue at work modified the association between night shift work and breast cancer among women aged <50 and ≥50 years. In addition, the use of median splits within a relatively small exposed subgroup may obscure or distort the underlying exposure–risk, dose–response relationship. Fourth, we were unable to adjust for several potential confounders, including age at first childbirth, first degree family history of breast or ovarian cancer, use of oral contraceptives, and hormone replacement therapy, which may have influenced the observed associations. Fifth, data on fatigue and chronotype were available for only approximately half of the study population, resulting in reduced statistical power and potentially limiting the generalizability of findings related to these variables. Sixth, the possibility of chance findings cannot be entirely ruled out due to the issue of multiple testing. Given the wide range of exposures and subgroup analyses conducted, it is statistically expected that a proportion – approximately 5–10% – of the observed significant or borderline significant associations may have occurred by chance. Therefore, especially findings that are not supported by prior evidence should be interpreted with caution. Finally, although payroll records help reduce recall bias and generally provide objective information on working hours, they are still susceptible to potential sources of error, such as recording mistakes, coding inconsistencies, or data-transfer issues, which may introduce some degree of exposure misclassification. However, based on the original validation of the method, the retrieved data did not contain any incorrect, missing, or outdated working-hour entries (20).

### Concluding remarks

This study showed that permanent night shift work is associated with an increased risk of breast cancer among women who report sleep problems or fatigue at work. The risk is particularly elevated among women aged ≥50 years working sequences of ≥3 consecutive night shifts. Psychological distress and chronotype did not consistently modify the association between night shift work and breast cancer risk.

Given the observational design, these findings cannot establish causality, but they provide important evidence that both shift scheduling and individual symptoms related to sleep and fatigue may be relevant when evaluating potential health risks associated with night shift work. Further studies, including intervention designs, are needed to determine whether modifying work schedules or addressing sleep problems and fatigue could influence breast cancer risk.

## Supplementary material

Supplementary material

## Data Availability

Data supporting this study cannot be made available due to ethical and legal restrictions.
