# Supplementary material for: Night shift work and breast cancer risk: A cohort study based on payroll and survey data from Finland
Source: Scand J Work Environ Health. 2026 Jun 26;52(4):452–61. doi: 10.5271/sjweh.4309 (PMC13349412; doi:10.5271/sjweh.4309)
Supplement: Supplementary material [file SJWEH-52-452-S001.pdf]

Night shift work and breast cancer risk: A cohort study based on payroll and survey data from Finland[1]

by Rahman Shiri,[2] Päivi Vanttola, Jenni Ervasti, Aki Koskinen, Johnni Hansen, Mikko Härmä

1. Supplementary material
2. Correspondence to: Rahman Shiri, Finnish Institute of Occupational Health, P.O. Box 18, FI-00032 Työterveyslaitos, Helsinki. [E-mail: rahman.shiri@ttl.fi] ORCID ID: 0000-0002-9312-3100

Supplemetary Table S1: The associations of shift and night work characteristics at baseline with breast cancer risk in women younger than 50 years and those aged 50 or older

Characteristic	Women younger than 50				Women aged 50 or older			
	Sample	Cancer cases	HR *	95% CI	Sample	Cancer cases	HR *	95% CI
Type of shift work, self-reported								
Regular day work	11 029	182	1		7087	267	1	
Shift work without night shifts	7034	83	0.93	0.70-1.22	4160	159	1.05	0.85-1.31
Shift work with night shifts	7378	77	0.73	0.54-0.996	2783	120	1.10	0.86-1.41
Permanent night work	518	8	0.92	0.42-2.01	264	14	1.27	0.69-2.35
Other irregular work	641	3	0.31	0.10-0.94	455	18	0.91	0.53-1.57
Number of non-day shifts								
None	8489	141	1		5526	202	1	
Below median	9039	123	0.84	0.65-1.10	4996	178	1.01	0.81-1.26
Above median	9613	91	0.70	0.52-0.94	4435	205	1.25	1.01-1.55
Number of morning shifts								
Low	9552	95	1		4492	184	1	
Medium	9112	119	1.15	0.85-1.54	4902	177	0.88	0.71-1.10
High	8477	141	1.36	1.01-1.82	5563	224	0.94	0.76-1.16
Number of evening shifts								
None	11 635	188	1		7845	291	1	
Below median	7860	100	0.87	0.67-1.13	3455	127	1.03	0.81-1.32
Above median	7646	67	0.71	0.52-0.96	3657	167	1.28	1.03-1.59
Number of night shifts >8 h								
None	17 200	251	1		11 426	427	1	
Below median	4885	53	0.81	0.59-1.10	1847	84	1.06	0.81-1.39
Above median	5056	51	0.84	0.59-1.20	1684	74	1.13	0.86-1.48
Number of night shifts >10 h								
None	18 435	264	1		12 046	452	1	
Below median	4223	54	0.96	0.69-1.33	1588	67	0.99	0.74-1.33
Above median	4483	37	0.73	0.50-1.05	1323	66	1.24	0.93-1.64
Number of night shifts >12 h								
None	22 711	308	1		13 506	531	1	
Below median	2205	25	0.96	0.63-1.47	719	25	0.78	0.50-1.22
Above median	2225	22	0.86	0.56-1.33	732	29	0.98	0.66-1.44
Number of consecutive night shifts								
None	17 178	251	1		11 414	427	1	
Below median	4302	50	0.77	0.56-1.06	1712	72	0.92	0.68-1.24
Above median	5661	54	0.87	0.62-1.22	1831	86	1.28	0.99-1.65
≥3 consecutive night shift								
No	21 163	308	1		13 134	501	1	
Yes	5978	47	0.71	0.49-1.02	1823	84	1.27	0.98-1.64
≥3 consecutive night shift								
None	21 163	308	1		13 134	501	1	
Below median	2981	23	0.69	0.45-1.06	787	32	0.85	0.54-1.32
Above median	2997	24	0.73	0.44-1.22	1036	52	1.64	1.22-2.21
≥5 consecutive night shift								

Characteristic	Women younger than 50				Women aged 50 or older			
	Sample	Cancer cases	HR *	95% CI	Sample	Cancer cases	HR *	95% CI
No	25 735	342	1		14 417	560	1	
Yes	1406	13	0.77	0.36-1.64	540	25	1.46	0.95-2.26
Shift intervals of <11 hours								
None	11 573	186	1		7715	289	1	
Below median	7900	85	0.77	0.57-1.03	3503	145	1.19	0.95-1.50
Above median	7668	84	0.80	0.61-1.06	3739	151	1.04	0.83-1.29
Number of recovery periods <28h after the last night shift								
None	23 762	323	1		13 778	532	1	
Below median	1764	17	0.73	0.44-1.20	626	24	1.02	0.67-1.56
Above median	1790	18	0.70	0.42-1.16	606	31	1.24	0.85-1.82
Number of recovery periods <48h after the last night shift								
None	21 444	302	1		13 033	497	1	
Below median	2836	27	0.76	0.51-1.12	1005	41	0.95	0.68-1.35
Above median	3036	29	0.69	0.45-1.04	972	49	1.17	0.85-1.61
Ratio of night shifts to all shifts								
None	17 178	251	1		11414	427	1	
Below median	4895	53	0.80	0.59-1.10	1855	84	1.05	0.80-1.38
Above median	5068	51	0.84	0.59-1.20	1688	74	1.12	0.85-1.48
Worktime control								
Low	4871	65	1		2970	96	1	
Medium	7522	87	0.93	0.66-1.29	3329	102	0.83	0.62-1.10
High	7258	103	1.23	0.89-1.71	2612	96	1.05	0.80-1.39

* Adjusted for age, marital status, living with a child, socioeconomic status, smoking, alcohol consumption, body mass index, leisure-time physical activity, job demands, job control, and worktime control

Supplemetary Table S2: The associations of shift and night work characteristics at baseline with breast cancer risk in women with and without sleep problems

Characteristic	Without sleep problems				With sleep problems			
	**Sample**	**Cancer cases**	**HR ***	**95% CI**	**Sample**	**Cancer cases**	**HR ***	**95% CI**
Number of non-day shifts								
None	6960	167	1		7274	179	1	
Below median	6714	140	0.94	0.74-1.21	7504	163	0.93	0.74-1.17
Above median	7167	139	0.91	0.71-1.18	7237	160	1.05	0.83-1.34
Number of morning shifts								
Low	6881	130	1		6821	147	1	
Medium	6509	134	1.04	0.80-1.37	7290	159	0.93	0.74-1.18
High	6693	174	1.20	0.93-1.55	7146	188	1,01	0.81-1.28
Number of evening shifts								
None	9812	241	1		9977	242	1	
Below median	5533	103	0.94	0.73-1.20	6000	126	0.97	0.76-1.24
Above median	5496	102	0.89	0.68-1.16	6038	134	1.12	0.88-1.42
Number of night shifts >8 h								
None	13 923	321	1		15 119	363	1	
Below median	3267	57	0.80	0.58-1.09	3621	82	1.08	0.83-1.40
Above median	3651	68	1.08	0.80-1.47	3275	57	0.88	0.66-1.18
Number of night shifts >10 h								
None	14 842	336	1		16 086	386	1	
Below median	2857	57	1.00	0.73-1.38	3089	65	0.96	0.74-1.26
Above median	3142	53	0.98	0.70-1.35	2840	51	0.96	0.70-1.31
Number of night shifts >12 h								
None	17 716	392	1		19 130	454	1	
Below median	1523	26	0.88	0.56-1.39	1461	25	0.84	0.56-1.28
Above median	1602	28	1.16	0.78-1.71	1424	23	0.72	0.46-1.12
Number of consecutive night shifts								
None	13 907	321	1		15 101	363	1	
Below median	3084	56	0.80	0.58-1.10	3072	68	0.90	0.68-1.20
Above median	3850	69	1.07	0.79-1.44	3842	71	1.07	0.81-1.41
≥3 consecutive night shift								
No	16 723	373	1		18 133	444	1	
Yes	4118	73	1.15	0.86-1.53	3882	58	0.82	0.61-1.11
≥3 consecutive night shift								
None	16 723	373	1		18 133	444	1	
Below median	1974	29	0.86	0.56-1.33	1909	26	0.68	0.43-1.06
Above median	2144	44	1.42	0.99-2.02	1973	32	0.97	0.66-1.43
≥5 consecutive night shift								
No	19 768	427	1		21 116	483	1	
Yes	1073	19	1.06	0.58-1.93	899	19	1.21	0.73-2.01
Shift intervals of <11 hours								
None	9655	237	1		9925	241	1	
Below median	5582	106	0.96	0.75-1.24	6024	127	1.01	0.79-1.29
Above median	5604	103	0.84	0.65-1.08	6066	134	1.01	0.80-1.27
Number of recovery periods <28h after the last night shift								
None	18 410	403	1		19 791	459	1	
Below median	1259	23	1.09	0.71-1.69	1186	18	0.68	0.41-1.13
Above median	1274	22	0.95	0.60-1.49	1164	28	0.98	0.65-1.48
Number of recovery periods <48h after the last night shift								

Characteristic	Without sleep problems				With sleep problems			
	Sample	Cancer cases	HR *	95% CI	Sample	Cancer cases	HR *	95% CI
None	16 800	373	1		18 272	432	1	
Below median	2057	38	0.98	0.67-1.43	1869	31	0.75	0.51-1.12
Above median	2086	37	0.98	0.69-1.39	2000	42	0.88	0.62-1.25
Ratio of night shifts to all shifts								
None	13 907	321	1		15 101	363	1	
Below median	3273	57	0.79	0.58-1.09	3633	82	1.06	0.82-1.38
Above median	3661	68	1.08	0.79-1.46	3281	57	0.88	0.66-1.17
Worktime control								
Low	3425	64	1		4419	97	1	
Medium	5332	87	0.91	0.66-1.27	5522	102	0.85	0.64-1.13
High	5213	100	1.21	0.88-1.66	4664	99	1.12	0.84-1.49

* Adjusted for age, marital status, childbirth, socioeconomic status, smoking, alcohol consumption, body mass index, leisure-time physical activity, job demands, job control, and worktime control

Supplemetary Table S3: The associations of shift and night work characteristics at baseline with breast cancer risk in women with and without fatigue at work

Characteristic	Without fatigue at work				With fatigue at work			
	Sample	Cancer cases	HR *	95% CI	Sample	Cancer cases	HR *	95% CI
Number of non-day shifts								
None	2990	105	1		4677	132	1	
Below median	3007	87	0.84	0.63-1.13	4172	117	1.11	0.84-1.46
Above median	3081	65	0.67	0.48-0.94	4565	135	1.27	0.97-1.65
Number of morning shifts								
Low	3110	67	1		4504	123	1	
Medium	2865	81	1.08	0.77-1.52	4423	118	0.94	0.73-1.23
High	3103	109	1.29	0.94-1.78	4487	143	0.98	0.76-1.27
Number of evening shifts								
None	4253	134	1		6476	192	1	
Below median	2717	75	1.03	0.77-1.38	3611	96	1.06	0.80-1.39
Above median	2108	48	0.79	0.55-1.13	3327	96	1.13	0.87-1.47
Number of night shifts >8 h								
None	5827	186	1		8708	268	1	
Below median	1546	38	0.86	0.59-1.24	2385	63	1.04	0.77-1.41
Above median	1705	33	0.79	0.53-1.17	2321	53	1.00	0.74-1.36
Number of night shifts >10 h								
None	6272	197	1		9305	284	1	
Below median	1331	32	0.93	0.63-1.38	1987	58	1.13	0.84-1.51
Above median	1475	28	0.76	0.51-1.15	2122	42	0.96	0.70-1.34
Number of night shifts >12 h								
None	7648	227	1		11 476	342	1	
Below median	718	11	0.61	0.32-1.15	1006	24	0.99	0.65-1.50
Above median	712	19	1.12	0.72-1.75	932	18	0.80	0.50-1.28
Number of consecutive night shifts								
None	5815	186	1		8695	268	1	
Below median	1621	37	0.75	0.52-1.09	2427	61	0.95	0.70-1.29
Above median	1642	34	0.90	0.61-1.33	2292	55	1.11	0.83-1.49
≥3 consecutive night shift								
No	7329	223	1		10 999	334	1	
Yes	1749	34	0.89	0.60-1.31	2415	50	0.96	0.71-1.30
≥3 consecutive night shift								
None	7329	223	1		10 999	334	1	
Below median	991	19	0.83	0.49-1.38	1388	24	0.79	0.51-1.23
Above median	758	15	0.97	0.55-1.69	1027	26	1.17	0.79-1.73
≥5 consecutive night shift								
No	8747	251	1		12 985	371	1	
Yes	331	6	0.84	0.34-2.07	429	13	1.31	0.75-2.29
Shift intervals of <11 hours								
None	4116	139	1		6275	182	1	
Below median	2408	63	0.89	0.65-1.20	3150	92	1.16	0.88-1.53
Above median	2554	55	0.68	0.50-0.94	3989	110	1.07	0.83-1.39
Number of recovery periods <28h after the last night shift								
None	7841	232	1		11 789	346	1	
Below median	622	10	0.63	0.33-1.21	859	21	1.03	0.65-1.63
Above median	631	15	0.89	0.52-1.51	797	19	0.94	0.57-1.54
Number of recovery periods <48h after the last night shift								
None	7082	213	1		10 684	321	1	
Below median	969	23	0.92	0.57-1.49	1430	29	0.87	0.59-1.27
Above median	1043	21	0.80	0.51-1.27	1331	36	1.06	0.74-1.53
Ratio of night shifts to all shifts								

Characteristic	Without fatigue at work				With fatigue at work			
	Sample	Cancer cases	HR *	95% CI	Sample	Cancer cases	HR *	95% CI
None	5815	186	1		8 695	268	1	
Below median	1558	38	0.85	0.59-1.23	2390	63	1.04	0.77-1.40
Above median	1705	33	0.79	0.53-1.17	2329	53	1.00	0.74-1.35
Worktime control								
Low	1152	29	1		2653	71	1	
Medium	2130	38	0.74	0.46-1.21	3282	72	0.84	0.61-1.17
High	2390	66	1.21	0.78-1.87	2361	58	1.03	0.73-1.47

* Adjusted for age, marital status, living with a child, socioeconomic status, smoking, alcohol consumption, body mass index, leisure-time physical activity, job demands, job control, and worktime control

Supplemetary Table S4: The associations of shift and night work characteristics at baseline with breast cancer risk in women with morning and evening chronotypes

Characteristic	Morning chronotype				Evening chronotype			
	Sample	Cancer cases	HR *	95% CI	Sample	Cancer cases	HR *	95% CI
Type of shift work, self-reported								
Regular day work	5648	130	1		4112	90	1	
Shift work without night shifts	2952	63	1.08	0.79-1.46	2801	44	0.98	0.66-1.44
Shift work with night shifts	2263	36	0.72	0.48-1.09	2987	60	0.96	0.66-1.40
Permanent night work	148	2	0.75	0.18-3.08	309	4	0.63	0.20-1.99
Other irregular work	286	7	1.07	0.50-2.31	254	3	0.54	0.17-1.64
Number of non-day shifts								
None	4394	96	1		3012	74	1	
Below median	4178	88	0.99	0.74-1.33	3163	50	0.69	0.47-1.01
Above median	2802	54	0.99	0.70-1.42	4384	76	0.78	0.55-1.11
Number of morning shifts								
Low	2830	49	1		4521	72	1	
Medium	4041	81	1.09	0.77-1.56	3178	60	1.23	0.86-1.77
High	4503	108	1.15	0.81-1.64	2860	68	1.34	0.93-1.93
Number of evening shifts								
None	5872	129	1		4292	87	1	
Below median	3094	63	0.98	0.72-1.35	2876	53	0.98	0.68-1.43
Above median	2408	46	1.02	0.72-1.45	3391	60	1.02	0.71-1.47
Number of night shifts >8 h								
None	8427	187	1		6333	125	1	
Below median	1691	31	0.81	0.54-1.20	1886	35	0.94	0.64-1.38
Above median	1256	20	0.85	0.54-1.36	2340	40	0.95	0.63-1.44
Number of night shifts >10 h								
None	8856	192	1		6889	138	1	
Below median	1367	25	0.85	0.56-1.31	1719	33	0.94	0.62-1.43
Above median	1151	21	0.94	0.58-1.50	1951	29	0.87	0.56-1.35
Number of night shifts >12 h								
None	10 126	216	1		8703	170	1	
Below median	641	10	0.82	0.44-1.54	950	18	1.02	0.60-1.73
Above median	607	12	0.97	0.53-1.78	906	12	0.72	0.39-1.32
Number of consecutive night shifts								
None	8416	187	1		6330	125	1	
Below median	1499	23	0.66	0.43-1.03	1753	30	0.80	0.52-1.22
Above median	1459	28	1.03	0.68-1.56	2476	45	1.08	0.76-1.55
≥3 consecutive night shift								
No	9890	211	1		7938	161	1	
Yes	1484	27	1.04	0.68-1.59	2621	39	0.89	0.61-1.31
≥3 consecutive night shift								
None	9890	211	1		7938	161	1	
Below median	796	12	0.84	0.46-1.53	1254	18	0.85	0.51-1.42
Above median	688	15	1.29	0.75-2.21	1367	21	0.93	0.57-1.51
≥5 consecutive night shift								
No	11 078	233	1		9869	186	1	
Yes	296	5	0.82	0.30-2.22	690	14	1.31	0.71-2.38
Shift intervals of <11 hours								
None	5880	131	1		4333	89	1	
Below median	2911	57	1.00	0.72-1.38	3081	51	0.91	0.63-1.31
Above median	2583	50	0.92	0.65-1.29	3145	60	0.98	0.69-1.41
Number of recovery periods <28h after the last night shift								
None	10 328	217	1		9066	180	1	
Below median	495	10	1.06	0.57-1.97	832	13	0.71	0.39-1.32

Characteristic	Morning chronotype				Evening chronotype			
	Sample	Cancer cases	HR *	95% CI	Sample	Cancer cases	HR *	95% CI
Above median	570	11	0.79	0.41-1.55	681	9	0.62	0.30-1.25
Number of recovery periods <48h after the last night shift								
None	9651	210	1		8091	161	1	
Below median	796	13	0.78	0.43-1.41	1298	24	0.89	0.56-1.41
Above median	946	15	0.67	0.38-1.18	1190	17	0.71	0.42-1.21
Ratio of night shifts to all shifts								
None	8416	187	1		6330	125	1	
Below median	1697	31	0.80	0.54-1.19	1883	35	0.94	0.64-1.37
Above median	1261	20	0.85	0.54-1.35	2346	40	0.95	0.63-1.43
Worktime control								
Low	3033	57	1		2790	64	1	
Medium	4304	84	1.00	0.71-1.40	4091	71	0.75	0.53-1.07
High	4050	97	1.34	0.96-1.86	3688	66	0.87	0.62-1.22

* Adjusted for age, marital status, living with a child, socioeconomic status, smoking, alcohol consumption, body mass index, leisure-time physical activity, job demands, job control, and worktime control
